# Palmitoleate attenuates diet induced insulin resistance and hepatic inflammation independently of PPAR-α

**DOI:** 10.1186/2049-3002-2-S1-P52

**Published:** 2014-05-28

**Authors:** Camila O Souza, Alexandre A de S Teixeira, Edson A de Lima, Helena A Batatinha, Sandro M Hirabara, William T Festuccia, José Cesar Rosa Neto

**Affiliations:** 1 Department of Cell and Developmental Biology, University of São Paulo, São Paulo, São Paulo, 05508-000, Brazil; 2 Institute of Physical Activity Sciences and Sports, Cruzeiro do Sul University, São Paulo, São Paulo, 01506-000, Brazil; 3 Department of Physiology and Biophysics, University of São Paulo, São Paulo, São Paulo, 05508-000, Brazil

## Background

Previous studies have showed that the lipokine palmitoleic acid (or palmitoleate) reduces diet induced muscle and liver inflammation and insulin resistance [1] and increases adipose tissue lipolysis, the latter through a mechanism that depends on PPAR-α [2]. Here we tested whether palmitoleate protects from the deleterious effects of a high fat diet (HFD) on glucose homeostasis and liver inflammation and whether PPAR-α is involved in these actions.

## Materials and methods

C57BL (C57) and PPAR-α knockout (KO) mice fed either with a balanced (BD) or HFD during 12 weeks were treated after the 10th week with oleic acid (OLA, 300mg/kg of b.w.) or palmitoleate (PMA, 300mg/kg of b.w.) and evaluated for glucose and insulin tolerances (GTT and ITT), glucose uptake and metabolism, serum levels of aspartate aminotransferase (AST) and hepatic triacylglycerol content and cytokines levels (ELISA) and gene expression (qRT-PCR).

## Results

HFD promotes insulin resistance and hepatic steatosis in both C57 and KO as evidenced by the reduced (p<0.05) muscle insulin stimulated glucose uptake and incorporation and increased (p<0.05) hepatic triacylglycerol content and plasma AST levels. Surprisingly, the hepatic steatosis induced by HFD was associated with liver inflammation in KO, but not C57 mice as shown by the increased (p<0.05) hepatic levels of IL1-β, IL-12 and TNF-α. In spite of these genotype specific phenotypes, HFD increased (p<0.05) TLR4 expression and decreased (p<0.05) the IL1-Ra expression similarly in both C57 and KO. Independently of mice genotype, palmitoleate markedly attenuated the insulin resistance induced by HFD as evidenced by the improved glucose tolerance and response to insulin in the ITT (p<0.05) and increased glucose uptake and incorporation in muscle in vitro (p<0.05). Furthermore, palmitoleate reduced (p<0.05) the serum levels of AST in C57 and decreased (p<0.05) the hepatic levels of IL1-β and IL-12 in KO mice. Finally, palmitoleate reduced the hepatic TLR-4 expression (p<0.05) and increased IL-1Ra expression (p<0.05) in C57 but not in KO.

## Conclusions

We concluded that palmitoleate attenuates diet induced insulin resistance, hepatic steatosis, inflammation and damage through mechanisms that do not depend on PPAR-α.
